# Transitions in smartphone addiction proneness among children: The effect of gender and use patterns

**DOI:** 10.1371/journal.pone.0217235

**Published:** 2019-05-30

**Authors:** Jeng-Tung Chiang, Fong-Ching Chang, Kun-Wei Lee, Szu-Yuan Hsu

**Affiliations:** 1 Department of Statistics, National Chengchi University, Taipei, Taiwan; 2 Department of Health Promotion and Health Education, National Taiwan Normal University, Taipei, Taiwan; Universita Cattolica del Sacro Cuore Sede di Roma, ITALY

## Abstract

**Objectives:**

This study assessed the incidence of transitions in smartphone addiction proneness (SAP) among children and examined the effects of gender, use patterns (social networking sites (SNSs) use and smartphone gaming) and depression on smartphone addiction transitions.

**Methods:**

A representative sample of 2,155 children from Taipei completed longitudinal surveys in both 2015 (5th grade) and 2016 (6th grade). Latent transition analysis (LTA) was used to characterize transitions in SAP and to examine the effects of gender, use patterns and depression on SAP transitions.

**Results:**

LTA identified four latent statuses of SAP: about half of the children were in non-SAP status, one-fifth were in tolerance status, one-sixth were in withdrawal status, and one-seventh were in high-SAP status. Both boys and girls had a higher prevalence of high-SAP and tolerance in 6^th^ grade than in 5^th^ grade, whereas in both grades boys had a higher prevalence of high-SAP and withdrawal, and girls had a higher prevalence of non-SAP and tolerance. Controlling for parents’ education, family structure, and household income, higher use of SNSs by children, increasing use of mobile gaming and higher levels of depression were individually associated with increased odds of being in one of the three SAP statuses other than non-SAP. When all three covariates were jointly entered into the model, usage of SNSs and depression remained significant predictors.

**Conclusion:**

Both boys and girls tended to transition to tolerance or high-SAP statuses, while children’s depression and their usage of SNSs increased the risk of smartphone addiction.

## Introduction

Smartphones have become a part of life for many children. Children spend more time on new media than on traditional media, and frequently use mobile devices to watch videos and for online gaming, searching, social networking, and instant messaging [1]. Although most Social Networking Sites (SNSs) (e.g., Facebook) state that the minimum age requirement for creating an account is 13, more children are using social networking sites (SNSs) and instant messaging. The prevalence of smartphone addiction proneness (SAP) among children and adolescents is rapidly increasing, particularly in Asian countries. A Korean study showed that 31% of middle school students had SAP [2]. Studies found that children’s excessive use of mobile devices had negative health impacts such as dry eye disease [3], myopia [4], sleep deprivation [5], anxiety [6], problem behaviors, and lower levels of emotional intelligence [7].

Children and adolescents undergo stages of rapid psychosocial development. Boys and girls of the same age are at different stages of puberty development and may be vulnerable to different online activities, risks and harm. The Australian Child and Adolescent Survey showed that girls had very high levels of psychological distress and problem behaviors that were twice that for boys of the same age. These problem behaviors were associated with frequent Internet use and electronic gaming [8]. Prior studies have found that male students were more prone to Internet addiction, while females were more prone to smartphone addiction [9, 10]. Another study associated game apps use with smartphone addiction among males, while the use of SNSs has been associated with smartphone addiction among women [11].

Smartphone addiction is characterized by symptoms of compulsive behavior, withdrawal, tolerance, and functional impairment [12, 13]. However, smartphone addiction is not included in the Diagnostic and Statistical Manual for Mental Disorders (DSM-5), while Internet Gaming Disorder (IGD) has been incorporated into the DSM-5 appendix (Section 3) as requiring additional research [14]. Some arguments regarding smartphone addiction have addressed the need to assess use patterns and activities to explore the features of smartphone addiction. Studies have associated smartphone use patterns such as SNSs and game use with smartphone addiction [2, 15–17]. In addition, studies have associated psychological symptoms such as depression and anxiety with smartphone addiction [11, 18–20].

Studies have applied different methods to explore the features and psychophysiological effects of SAP, and debate is ongoing as to whether the descriptive term “smartphone addiction” is more appropriate than “problem smartphone use” [21]. Some studies have used techniques such as a telemetric approach and smartphone applications to objectively measure smartphone use, time, and activity [22–25], while at least one study has used wearable sensors that detect autonomic activation to measure psychophysiological effects [26]. Another study conducted gambling/gaming task tests and skin conductance responses and found that smartphone addiction groups displayed impairments in the decision-making process, which were similar to those found in chemical addictions [27].

Smartphone addiction is an emerging public health issue. The epidemic of smartphone addiction has rapidly spread from young adults and adolescents to children in many countries. Taiwan news reported a 10-year-old boy died after his parent took away his smartphone. However, Taiwan has officially recognized e-sports as an official competitive sport, while more politicians have endorsed e-sports markets. Experts and parents worry that the negative impact of these events might cause more children and adolescents to develop Internet and smartphone addictions. Studies have developed scales to measure smartphone addiction, but most of these studies are based on young adults and adolescents. Experts have criticized the different scales and cut-point values that may cause variations in the definition and features of smartphone addiction. Little research has been focused on assessing transitions in smartphone addiction among children and examining the effects of gender, use patterns and depression on smartphone addiction transitions. Latent Class Analysis (LCA) has proven to be highly useful in recent years for modeling complex behavior, while Latent Transition Analysis (LTA), a longitudinal extension of LCA, could be used to model transition patterns and developments in discrete latent variables [28]. This study used LTA to identify the latent statuses of SAP and transitions in SAP from 5th grade to 6th grade. Effects of gender and smartphone use patterns were also explored.

## Methods

### Participants and procedures

In 2015, a total of 320,441 students attended 367 primary schools in Taipei City and New Taipei City, Taiwan. Based on the sampling frame, which was a list of schools and their student enrollments, a probability-proportionate-to-size sampling method was used to systematically draw a random sample of schools. A total of 47 schools were invited to join the survey, and 30 schools agreed to participate. Three to five classes were randomly selected from each sample school. Approval was obtained from the Institutional Review Board at National Taiwan Normal University.

Following class selection, teachers gave students consent forms to take home to parents requesting consent for their children to participate in the survey. The children also filled out a consent form to indicate their willingness to participate in this study. Researchers visited the schools to conduct a self-administered survey. A total of 2,621 fifth-grade students from 30 sample schools completed the questionnaire. The response rate was 86%.

In 2016, when the students were in the 6th grade, participating students were re-contacted to complete the same questionnaire. A total of 2,155 students completed the questionnaire in both the 2015 and 2016 surveys. About 17.8% of students dropped out of the follow-up survey, because some students refused to participate and some transferred to other schools or were absent on that day. The responses of the 2,155 students who completed both surveys were used to examine the changes and relationships between smartphone use patterns, depression, and smartphone addiction.

### Instrument

A self-administered questionnaire was developed based on previous studies [29, 30]. A group of 8 experts were invited to assess the content validity of the questionnaire. Experts reviewed the draft questionnaire and provided comments for improvement. In addition, a pilot survey was conducted at two schools that were not included in the sample schools in order to examine the students’ responses to the survey and to evaluate the reliability of the data that the questionnaire would yield.

### Smartphone addiction

Smartphone addiction was assessed using a short-form Smartphone Addiction Inventory [31]. Six items of the smartphone addiction scale were chosen to assess the symptoms of smartphone addiction. For example, participants were asked whether they agreed or disagreed with statements such as the following: “I use a smartphone for a longer period of time and spend more money than I had intended (compulsive behavior);” “I feel aches and soreness in the back or eye discomfort due to excessive smartphone use (functional impairment);” “I feel restless and irritable when the smartphone is unavailable (withdrawal);” and, “I find that I have been hooking on the smartphone longer and longer (tolerance).” Children’s reports of “disagree” or “strongly disagree” were coded as 1, while children’s reports of “strongly agree” or “agree” were coded as 2.

### Smartphone use patterns

Children were asked how many days during the past week they used a smartphone/tablet for social networking websites and/or instant messaging. The average of the responses on the two items was taken as a measure of their SNSs usage. Children were also asked how many days during the past week they used a smartphone/tablet for playing games alone and playing games with others. The average of the two responses was used to measure children’s game playing.

### Depression

Depression was measured using the Center for Epidemiologic Studies Depression Scale (CES-D) [32]. The CES-D is a 20-item scale that evaluates the presence of depressive symptoms. Children were asked during the past week how often they experienced feelings such as the following: “I was bothered by things that don’t usually bother me,” and “I did not feel like eating; my appetite was poor.” Response options for each item included the following: “rarely or none of the time (<1 day),” (scored 0); “some or a little of the time (1–2 days),” (scored 1); “occasionally or a moderate amount of the time (3– days),” (scored 2); or, “most or all of the time (5–7 days)” (scored 3). The total score of the scale ranged from 0 to 60. Cronbach’s α of the CES-D scale for children was 0.88.

### Controlled variables

Controlled variables included parental education, family structure, and household income. Children were asked questions regarding their parental educational attainment, family structure (parental marital status: married (coded 1); divorced/separated (coded 0)), and household income (lower-income class/lower-middle income class (coded 0); and, median/upper income class (coded 1)).

### Statistical analysis

SAS was used to perform the statistical analysis. PROC LTA was used to identify smartphone addiction statuses based on response patterns on smartphone addiction proneness items, and to estimate the prevalence of latent statuses, and transition probabilities from 2015 (Time 1) when students were in the 5^th^ grade to 2016 (Time 2) when they were in the 6th grade. Gender was considered as a group variable. In addition, SNSs usage, smartphone gaming, depression, and three controlled variables (parents’ education, family structure, and household income) were incorporated in the LTA to examine the effects of use patterns (SNSs usage and smartphone gaming) and level of depression on SAP latent statuses in the 5th grade by gender, and on transition probabilities from the 5^th^ grade to the 6^th^ grade.

## Results

### Characteristics of the participants

Participant children included 1,367 boys (52.2%) and 1,254 girls (47.8%). About 15% of the children reported that their parents were divorced or separated, while 25% of the children reported that their household income was of the lower or lower-middle income class.

### SAP latent status by gender

LTA models with two to five latent statuses were conducted to identify the SAP status number. Fit statistics, which includes the likelihood-ratio statistic G^2^, AIC and BIC, and degrees of freedom, all were cross-classified by measurement invariance imposition, and are summarized in Table 1. Note that measurement invariance across times simply means that the interpretation of the latent statuses is identical across times, and measurement invariance across groups means that the latent statuses can be identically interpreted across groups. As the table shows, regardless of whether or not the property of measurement invariance was imposed across times or across both times and groups, models with four latent statuses all had the lowest BICs, and had AICs that were only slightly larger than those with five latent statuses, and thus provided a balance of fit and parsimony. In addition, G^2^s that were associated with the three four-status models were not statistically significantly different ($G_{1}^{2}-G_{2}^{2}=23.48,$ df_1_−df_2_ = 24, p-value = 0.51; $G_{2}^{2}-G_{3}^{2}=49.18,$ df_2_−df_3_ = 48, p-value = 0.57; $G_{1}^{2}-G_{3}^{2}=72.66,$ df_1_−df_3_ = 72, p-value = 0.54). Measurement invariance across both times and groups appeared reasonable.

**Table 1:** Model fit information for selecting the number of latent statuses of SAP.

Model	Measurement invariance	Number of latent statuses	G^2^	df	AIC	BIC
	Times, Groups	Two	3944.58	8173	3980.58	4080.87
		Three	3244.59	8157	3312.59	3502.02
1		**Four**	**2876.86**	**8137**	**2984.86**	**3285.72**
		Five	2801.88	8113	2957.88	3392.45
	Times only	Two	3916.78	8161	3976.78	4143.92
		Three	3217.09	8139	3321.09	3610.81
2		**Four**	**2853.38**	**8113**	**3009.38**	**3443.95**
		Five	2761.33	8083	2977.33	3579.05
	None	Two	3874.10	8137	3982.10	4282.96
		Three	3164.25	8103	3340.25	3830.54
3		**Four**	**2804.20**	**8065**	**3056.20**	**3758.21**
		Five	2685.89	8023	3021.89	3957.90

Note. G^2^: Likelihood-ratio statistic, AIC: Akaike Information Criterion

BIC: Bayesian Information Criterion, Times: 2015(grade 5), 2016(grade 6)

Groups: boys, girls

Given that measurement invariance has been established, it is then legitimate to address the question of whether the prevalence of latent statuses at Time 1 are equal across groups and/or the question of whether the transition probabilities are equal across groups. Table 2 presents the model fit statistics that are associated with the four latent transition models showing different types and degrees of equivalence across groups. As shown in the table, only Model 4, assuming that transition probabilities are equal across groups in addition to measurement invariance across both times and groups, was found to be statistically insignificantly different from Model 1, which was the model assuming only measurement invariance across both times and groups ($G_{4}^{2}-G_{1}^{2}=15.30,$ df_4_−df_1_ = 12, p-value = 0.2225). Model 5, in which the prevalence of latent statuses was constrained to be equal across gender at Time 1, and Model 6, in which both sets of parameters were constrained to be equal across gender, were both statistically significantly different from Model 1 ($G_{5}^{2}-G_{1}^{2}$ = 17.99, df_5_−df_1_ = 3, p-value = 0.0000; $G_{6}^{2}-G_{1}^{2}$ = 31.49, df_6_−df_1_ = 15, p-value = 0.0075). In addition, Model 4 also exhibited the lowest AIC value with a BIC that was only slightly larger than the lowest value. Hence, the four-status model with measurement invariance across both times and groups, and equal transition probabilities across groups was considered in the subsequent analyses.

**Table 2:** Fit statistics for test of gender differences in latent status prevalences and transition probabilities.

Model	Latent Status Prevalences at Time 1 equal across gender	Transition Probabilities equal across gender	G^2^	df	AIC	BIC
1	No	No	2876.86	8137	2984.86	3285.72
4	No	Yes	2892.16	8149	2976.16	3210.16
5	Yes	No	2894.85	8140	2996.85	3280.99
6	Yes	Yes	2908.35	8152	2986.35	3203.64

Note. G^2^: Likelihood-ratio statistic, AIC: Akaike Information Criterion

BIC: Bayesian Information Criterion, Time 1: 2015 (grade 5)

The item-response probabilities for observing an “agree” response on each item of SAP, conditional on each of the four latent statuses, are presented in the upper portion of Table 3. Because measurement invariance across times and groups was justified, the item-response probabilities were constrained to be equal across times and groups. Thus, only one set of these parameters is reported there. The first latent status was labeled non-SAP, because children in this status had a very low probability of reporting smartphone addiction proneness features. The second latent status was labeled tolerance because children in this status reported a high probability only of hooking on smartphones for longer periods of time. The third latent status was labeled withdrawal because children in this status tended to feel restless and irritable when the smartphone was unavailable, and, to a lesser degree, felt uneasy once stopping smartphone use for a certain period of time. The fourth latent status was labeled high SAP because children reported a high probability for every smartphone addiction proneness feature, which was more pronounced for tolerance and withdrawal features.

**Table 3:** Item-response probabilities, prevalence of latent statuses, and transition probabilities of SAP among boys and girls.

	Latent status			
Item-response probabilities to indicator of SAP	Non-SAP	Tolerance	Withdrawal	High-SAP
**Compulsive behavior**				
I use smartphone for a longer period of time and spend more money than I had intended.	0.0423	0.3247	0.1923	0.6349
**Functional impairment**				
To use smartphone has exercised certain negative effects on my schoolwork or job performance.	0.0866	0.2816	0.2482	0.5917
**Tolerance**				
I find that I have been hooking on smartphone longer and longer.	0.0833	0.8462	0.4563	0.9753
I have increased substantial amount of time using smartphone per week in recent three months.	0.0136	0.4491	0.1434	0.8259
**Withdrawal**				
I feel restless and irritable when the smartphone is unavailable.	0.0233	0.1373	0.7722	0.9335
I feel uneasy once I stop smartphone for a certain period of time.	0.0143	0.0412	0.5920	0.8742
**Prevalence of latent statuses**				
Boys				
5^th^ grade	0.4822	0.2114	0.1835	0.1229
6^th^ grade	0.4063	0.2664	0.1693	0.1581
Girls				
5^th^ grade	0.5095	0.2698	0.1127	0.1080
6^th^ grade	0.4195	0.2964	0.1350	0.1490
**Transition probabilities** **(Rows for 5**^**th**^ **grade, columns for 6**^**th**^ **grade)**				
Non-SAP	0.6614	0.2063	0.0858	0.0465
Tolerance	0.1770	0.6063	0.0437	0.1731
Withdrawal	0.1867	0.1316	0.5093	0.1724
High-SAP	0.1274	0.1188	0.2051	0.5486

Note. SAP: Smartphone addiction proneness

The middle portion of Table 3 shows the prevalence of latent statuses of SAP for boys and girls, respectively. When in the 5^th^ grade, compared with girls, boys were more likely to be in high-SAP (12.3% vs. 10.8%) and withdrawal (18.4% vs. 11.3%), and less likely to be in tolerance (21.1% vs. 27.0%) and low-SAP (48.2% vs. 51.0%). Because equal transition probabilities across groups were established, Table 3 also shows that the gender difference patterns associated with each latent status remained the same when students were in the 6^th^ grade.

### Transition of SAP by gender

The lower portion of Table 3 shows the transition probabilities. Because the transition probabilities were not statistically significantly different across gender, only one set of these parameters is presented. About half of the children remained at the same SAP status from 5^th^ grade to 6^th^ grade. One-fifth of those who were originally in the non-SAP status transitioned to the tolerance status (20.63%); of those originally in the tolerance status, 17.31% transitioned to the high-SAP status, whereas 17.70% transitioned to the non-SAP status; of those originally in the withdrawal status, 17.24% transitioned to the high-SAP status, whereas 18.67% transitioned to the non-SAP status; and, among those who were originally in the high-SAP status, 20.51% transitioned to the withdrawal status, while 12.74% even transitioned to non-SAP status. Overall, compared with when they were in the 5^th^ grade, in the 6^th^ grade the probability of being in the tolerance status or high-SAP status increased, whereas the probability of being in the SAP status or withdrawal status decreased.

### Effects of use patterns and depression on SAP

Controlling for parents’ education, family structure, and household income, the effects of use patterns (SNSs usage and smartphone gaming) and depression were explored for the effect on SAP latent statuses in the 5th grade by gender, and on transition probabilities from 5^th^ grade to 6^th^ grade. However, since equal transition probabilities across gender were concluded, Table 4 presents only the results of analyses using children’s SNSs usage, mobile gaming, and depression individually (Models 4.1–3) and jointly (Model 4.4) as primary covariates to predict membership in SAP latent statuses at Time 1 when students were in the 5^th^ grade. All three covariates were statistically significant predictors of SAP status for both boys and girls when assessed individually. On the other hand, when all three covariates were entered together in a model, with the effects of the other two covariates being partialed out, SNSs usage and depression remained statistically significant predictors, while mobile gaming was not due to the collinearity between SNSs usage and mobile gaming. As indicated in Model 4.4 of Table 4, other things being equal, for boys who reported one more day of SNS usage, the odds of being in tolerance relative to non-SAP became 1.0412 times higher, and the odds of being in withdrawal relative to non-SAP were 1.0534 times higher, while the odds of being in high-SAP relative to non-SAP were 1.0626 times higher. Compared with boys, the effect of SNS usage for girls was slightly larger. Relative to non-SAP, the odds of being in tolerance were 1.1175 times higher, the odds of being in withdrawal were 1.1297 times higher, and the odds of being in high-SAP were 1.1813 times higher. The effect of depression was similar.

**Table 4:** Odds ratios for the effects of use patterns and depression on SAP in the 5^th^ grade.

Odds ratios for the effects of use patterns and depression on SAP in the 5^th^ grade									
	Latent status for boys				Latent status for girls				Overall P-value
	Non-SAP	Tolerance	Withdrawal	High-SAP	Non-SAP	Tolerance	Withdrawal	High-SAP	
**Model 4.1**									
Mobile gaming	-	1.1779	1.1479	1.2452	-	1.1281	1.1674	1.2691	0.0000
**Model 4.2**									
SNSs using	-	1.1190	1.1236	1.1744	-	1.2460	1.2783	1.3962	0.0000
**Model 4.3**									
Depression	-	1.0170	1.0466	1.0458	-	1.0387	1.0126	1.0473	0.0000
**Model 4.4**									
Mobile gaming	-	1.0741	1.0564	1.1105	-	1.0279	1.0385	1.0751	0.9166
SNSs using	-	1.0412	1.0534	1.0626	-	1.1175	1.1297	1.1813	0.0000
Depression	-	1.0119	1.0353	1.0330	-	1.0082	1.0297	1.0367	0.0000

Note. N(Boy) = 1016, N(Girl) = 926

All models include parental education level, family structure, and household income as controlled variables.

## Discussion

This study identified four latent statuses of SAP: non-SAP, tolerance, withdrawal, and high-SAP. Both boys and girls had a higher prevalence of high-SAP and tolerance in the 6th grade than in the 5th grade. In either grade, boys had a higher prevalence of high-SAP and withdrawal, while girls had a higher prevalence of non-SAP and tolerance. Korean studies have found that boys have higher smartphone addiction risk behaviors [33], while girls tend to use mobile devices more frequently [6]. A German study found that girls were more prone to smartphone addiction [34]. Since boys and girls have different levels of puberty development, smartphone use patterns, and psychological distress prevalence, whether these factors or other determinants cause gender differences in the prevalence of SAP status will require further research. Further development and implementation of SAP prevention programs also are needed to reduce the levels of transition to more severe levels of SAP status among children.

In addition, this study found that children in high-SAP status had a more than 90% probability of reporting withdrawal and tolerance features. In another Korean study, adolescents with smartphone addiction had significantly higher scores on preoccupation, tolerance, lack of control, and withdrawal [35]. Other studies have also associated these features with smartphone addiction [36, 37]. These findings could provide insight for further screening and diagnostic criteria for smartphone addiction among children. The gender differences in some features of smartphone addiction suggest a need to further research other potential factors and design gender-specific intervention plans for smartphone addiction prevention among children. In addition, parents must be taught to identify whether their children have these SAP features and how to implement further preventive measures.

This study found that higher SNSs usage increased the odds of being in the other three SAP statuses relative to the non-SAP status for both genders. Prior studies have also associated greater use of SNSs with smartphone addiction among adolescents [2, 17] and among female college students [11]. One reason may be the high availability of smartphones to instant messaging and SNSs applications for interpersonal relationships [38]; whereas smartphone addiction may be part of SNS addiction [39]. A European study found that children who use SNSs encountered more online risks, while children with a public profile or many contacts encountered more risks [40]. Since SNSs (e.g., Facebook) state that the minimum age requirement for creating an account is 13, parents should talk to their children regarding the risks of SNSs and set rules to limit use in order to reduce online risks.

This study found that an increase in mobile gaming was also more likely to increase the odds of being in the other three SAP statuses relative to the non-SAP status. However, mobile gaming became an insignificant predictor when considered jointly with the use of SNSs due to the collinearity between the two covariates. Some studies have indicated that mobile gaming is a risk factor for smartphone addiction in adolescents [2], while at least one study associated game apps with smartphone addiction among male students [11]. Studies suggest that smartphone use patterns are crucial to assess and provide measures that can prevent and treat smartphone addiction [15, 16]. Clinical and public health professionals support the World Health Organization perspective of including gaming disorders in the 11th Revision of the International Classification of Diseases (ICD-11) in order to facilitate treatment and prevention [41]. Governments should institute comprehensive measures to prevent problematic online game use and smartphone addiction. The French government passed a law banning the use of smartphones in primary and middle schools to combat racketeering, theft, online bullying, and harassment in schools. Governments should also empower parents to implement effective strategies to prevent children’s smartphone addiction.

Moreover, this study found that an increase in a child's depression score is a significant predictor of smartphone addiction. Other studies [11, 18–20] have also found that depression was a risk factor for smartphone addiction. One Korean study found that children with a higher social network, as determined by the quality of the relationship with their parents and the size their peer group, showed a decrease in smartphone addiction [42]. To prevent the transition to a higher SAP, parents should invest more time in understanding their children’s online activities and the impact those activities are having on the mental health of their children. Schools should implement mental health promotion programs that will enhance children’s resilience and provide consultation to help SAP children and their parents.

This study had some limitations. First, 18% of the children dropped out of the follow-up survey. Second, social desirability bias may have had an influence on the truthfulness of the reports of smartphone addiction. However, confidentiality was emphasized, and trained investigators collected the questionnaires immediately. Finally, participant children might have difficulties recalling times spent on smartphone activities and reporting psychophysiological impact through self-administered questionnaires. Note that some studies applied novel methods to explore smartphone activity and SAP features. For example, one used wearable sensors measuring autonomic activation to detect smartphone addiction features and psychophysiological impact [26], while others used techniques such as telemetric approaches and smartphone applications to objectively measure screen time and smartphone activity [22–25]. Hence, future studies might consider using these techniques to better understand smartphone addiction features and psychophysiological effects of smartphone interaction on children and other populations.

## Conclusions

This is the first study using LTA to identify the transition patterns of smartphone addiction among children. The strength of this study is that a large sample of students (2155) was surveyed in grade 5 and was followed up a year later in grade 6 to explore the effects of smartphone use patterns and depression on transitions in smartphone addiction among children. This study identified four latent statuses of SAP: about half of the children were in non-SAP status, one-fifth were in tolerance status, one-sixth were in withdrawal status, and one-seventh were in high-SAP status. Boys had a higher prevalence of high-SAP and withdrawal, while girls had a higher prevalence of non-SAP and tolerance. Of 5^th^ grade children in non-SAP status, 21% had transitioned to tolerance by the 6^th^ grade; 9% had transitioned to withdrawal; and, 5% had transitioned to high-SAP. Both boys and girls had a higher prevalence of high-SAP and tolerance in the 6^th^ grade than in the 5^th^ grade. Controlling for parents’ education, family structure, and household income, for both boys and girls a higher rate of SNSs usage, mobile gaming and depression was individually associated with an increase in the odds of being in one of the three SAP statuses other than non-SAP. When all three covariates were jointly incorporated into the model, SNSs usage and depression remained significant predictors. Children’s SNSs usage and depression increased the risks of smartphone addiction.

## References

[1] Ofcom. Children and parents: media use and attitudes report2017.

[2] Cha SS, Seo BK. Smartphone use and smartphone addiction in middle school students in Korea: Prevalence, social networking service, and game use. Health psychology open. 2018;5(1):2055102918755046 Epub 2018/02/13. 10.1177/2055102918755046 ; PubMed Central PMCID: PMCPmc5802650. 29435355 PMC5802650

[3] Moon JH, Kim KW, Moon NJ. Smartphone use is a risk factor for pediatric dry eye disease according to region and age: a case control study. BMC ophthalmology. 2016;16(1):188 Epub 2016/10/30. 10.1186/s12886-016-0364-4 ; PubMed Central PMCID: PMCPmc5084437. 27788672 PMC5084437

[4] Saxena R, Vashist P, Tandon R, Pandey RM, Bhardawaj A, Menon V, et al Prevalence of myopia and its risk factors in urban school children in Delhi: the North India Myopia Study (NIM Study). PloS one. 2015;10(2):e0117349 Epub 2015/02/27. 10.1371/journal.pone.0117349 ; PubMed Central PMCID: PMCPmc4342249. 25719391 PMC4342249

[5] Brambilla P, Giussani M, Pasinato A, Venturelli L, Privitera F, Miraglia Del Giudice E, et al Sleep habits and pattern in 1–14 years old children and relationship with video devices use and evening and night child activities. Italian journal of pediatrics. 2017;43(1):7 Epub 2017/03/05. 10.1186/s13052-016-0324-x ; PubMed Central PMCID: PMCPmc5347825. 28257638 PMC5347825

[6] Kim R, Lee KJ, Choi YJ. Mobile Phone Overuse Among Elementary School Students in Korea: Factors Associated With Mobile Phone Use as a Behavior Addiction. Journal of addictions nursing. 2015;26(2):81–5. Epub 2015/06/09. 10.1097/JAN.0000000000000074 . 26053080

[7] Cho K-S, Lee J-M. Influence of smartphone addiction proneness of young children on problematic behaviors and emotional intelligence: Mediating self-assessment effects of parents using smartphones. Computers in Human Behavior. 2017;66:303–11. 10.1016/j.chb.2016.09.063 . PMC6387899 30834900

[8] Rikkers W, Lawrence D, Hafekost J, Zubrick SR. Internet use and electronic gaming by children and adolescents with emotional and behavioural problems in Australia—results from the second Child and Adolescent Survey of Mental Health and Wellbeing. BMC public health. 2016;16:399 Epub 2016/05/15. 10.1186/s12889-016-3058-1 ; PubMed Central PMCID: PMCPmc4866411. 27178325 PMC4866411

[9] Yang SY, Lin CY, Huang YC, Chang JH. Gender differences in the association of smartphone use with the vitality and mental health of adolescent students. Journal of American college health: J of ACH. 2018:1–9. Epub 2018/03/23. 10.1080/07448481.2018.1454930 . 29565784

[10] Mok JY, Choi SW, Kim DJ, Choi JS, Lee J, Ahn H, et al Latent class analysis on internet and smartphone addiction in college students. Neuropsychiatric disease and treatment. 2014;10:817–28. Epub 2014/06/06. 10.2147/NDT.S59293 ; PubMed Central PMCID: PMCPmc4038421. 24899806 PMC4038421

[11] Chen B, Liu F, Ding S, Ying X, Wang L, Wen Y. Gender differences in factors associated with smartphone addiction: a cross-sectional study among medical college students. BMC Psychiatry. 2017;17(1):341 Epub 2017/10/12. 10.1186/s12888-017-1503-z ; PubMed Central PMCID: PMCPmc5634822. 29017482 PMC5634822

[12] Lopez-Fernandez O. Short version of the Smartphone Addiction Scale adapted to Spanish and French: Towards a cross-cultural research in problematic mobile phone use. Addictive behaviors. 2017;64:275–80. Epub 2015/12/22. 10.1016/j.addbeh.2015.11.013 . 26685805

[13] Lin Y-H, Chiang C-L, Lin P-H, Chang L-R, Ko C-H, Lee Y-H, et al Proposed Diagnostic Criteria for Smartphone Addiction. PloS one. 2016;11(11):1–11. 10.1371/journal.pone.0163010 . PMC5112893 27846211

[14] Petry NM, Rehbein F, Ko CH, O'Brien CP. Internet Gaming Disorder in the DSM-5. Current psychiatry reports. 2015;17(9):72 Epub 2015/07/29. 10.1007/s11920-015-0610-0 . 26216590

[15] Liu CH, Lin SH, Pan YC, Lin YH. Smartphone gaming and frequent use pattern associated with smartphone addiction. Medicine. 2016;95(28):e4068 Epub 2016/07/20. 10.1097/MD.0000000000004068 ; PubMed Central PMCID: PMCPmc4956785. 27428191 PMC4956785

[16] Choi J, Rho MJ, Kim Y, Yook IH, Yu H, Kim DJ, et al Smartphone dependence classification using tensor factorization. PloS one. 2017;12(6):e0177629 Epub 2017/06/22. 10.1371/journal.pone.0177629 ; PubMed Central PMCID: PMCPmc5479529. 28636614 PMC5479529

[17] Haug S, Castro RP, Kwon M, Filler A, Kowatsch T, Schaub MP. Smartphone use and smartphone addiction among young people in Switzerland. Journal of behavioral addictions. 2015;4(4):299–307. Epub 2015/12/23. 10.1556/2006.4.2015.037 ; PubMed Central PMCID: PMCPmc4712764. 26690625 PMC4712764

[18] Demirci K, Akgonul M, Akpinar A. Relationship of smartphone use severity with sleep quality, depression, and anxiety in university students. Journal of behavioral addictions. 2015;4(2):85–92. Epub 2015/07/02. 10.1556/2006.4.2015.010 ; PubMed Central PMCID: PMCPmc4500888. 26132913 PMC4500888

[19] Matar Boumosleh J, Jaalouk D. Depression, anxiety, and smartphone addiction in university students- A cross sectional study. PloS one. 2017;12(8):e0182239 Epub 2017/08/05. 10.1371/journal.pone.0182239 ; PubMed Central PMCID: PMCPmc5544206. 28777828 PMC5544206

[20] Elhai JD, Dvorak RD, Levine JC, Hall BJ. Problematic smartphone use: A conceptual overview and systematic review of relations with anxiety and depression psychopathology. Journal of affective disorders. 2017;207:251–9. Epub 2016/10/14. 10.1016/j.jad.2016.08.030 . 27736736

[21] Panova T, Carbonell X. Is smartphone addiction really an addiction? Journal of behavioral addictions. 2018;7(2):252–9. Epub 2018/06/14. 10.1556/2006.7.2018.49 ; PubMed Central PMCID: PMCPmc6174603. 29895183 PMC6174603

[22] Christensen MA, Bettencourt L, Kaye L, Moturu ST, Nguyen KT, Olgin JE, et al Direct Measurements of Smartphone Screen-Time: Relationships with Demographics and Sleep. PloS one. 2016;11(11):e0165331 Epub 2016/11/10. 10.1371/journal.pone.0165331 27829040 PMC5102460

[23] McDonald CC, Ward K, Huang Y, Wiebe DJ, Delgado MK. Novel Smartphone-Based Measures of Cell Phone Use While Driving in a Sample of Newly Licensed Adolescent Drivers. Health Education & Behavior. 2019;46(1):10–4. EJ1202670. 30041576 10.1177/1090198118788612 PMC6345599

[24] Rod NH, Dissing AS, Clark A, Gerds TA, Lund R. Overnight smartphone use: A new public health challenge? A novel study design based on high-resolution smartphone data. PloS one. 2018;13(10):e0204811 Epub 2018/10/17. 10.1371/journal.pone.0204811 ; PubMed Central PMCID: PMCPmc6191085. 30325929 PMC6191085

[25] Prasad S, Harshe D, Kaur N, Jangannavar S, Srivastava A, Achanta U, et al A Study of Magnitude and Psychological Correlates of Smartphone Use in Medical Students: A Pilot Study with A Novel Telemetric Approach. Indian journal of psychological medicine. 2018;40(5):468–75. Epub 2018/10/03. 10.4103/IJPSYM.IJPSYM_133_18 ; PubMed Central PMCID: PMCPmc6149309. 30275623 PMC6149309

[26] Tonacci A, Billeci L, Sansone F, Masci A, Pala AP, Domenici C, et al An Innovative, Unobtrusive Approach to Investigate Smartphone Interaction in Nonaddicted Subjects Based on Wearable Sensors: A Pilot Study. Medicina (Kaunas, Lithuania). 2019;55(2). Epub 2019/02/06. 10.3390/medicina55020037 ; PubMed Central PMCID: PMCPmc6409719. 30720738 PMC6409719

[27] Khoury JM, Couto L, Santos DA, VHO ES, Drumond JPS, Silva L, et al Bad Choices Make Good Stories: The Impaired Decision-Making Process and Skin Conductance Response in Subjects With Smartphone Addiction. Frontiers in psychiatry. 2019;10:73 Epub 2019/03/12. 10.3389/fpsyt.2019.00073 ; PubMed Central PMCID: PMCPmc6395375. 30853918 PMC6395375

[28] Lanza ST, Collins LM. A new SAS procedure for latent transition analysis: transitions in dating and sexual risk behavior. Developmental psychology. 2008;44(2):446–56. Epub 2008/03/12. 10.1037/0012-1649.44.2.446 ; PubMed Central PMCID: PMCPmc2846549. 18331135 PMC2846549

[29] Mascheroni G, Ólafsson K. Net Children Go Mobile: risks and opportunities. Milano: Educatt; 2014.

[30] Livingstone S, Haddon L, Görzig A, Ólafsson K. The 2010 EU Kids Online Survey2011.

[31] Lin YH, Pan YC, Lin SH, Chen SH. Development of short-form and screening cutoff point of the Smartphone Addiction Inventory (SPAI-SF). International journal of methods in psychiatric research. 2017;26(2). Epub 2016/09/24. 10.1002/mpr.1525 . 27658956 PMC6877212

[32] Radloff LS. The CES-D scale: A self-report depression scale for research in the general population. Applied Psychological Measurement. 1977;1:385–401.

[33] Lee EJ, Kim HS. Gender Differences in Smartphone Addiction Behaviors Associated With Parent-Child Bonding, Parent-Child Communication, and Parental Mediation Among Korean Elementary School Students. Journal of addictions nursing. 2018;29(4):244–54. Epub 2018/12/07. 10.1097/JAN.0000000000000254 . 30507820

[34] Randler C, Wolfgang L, Matt K, Demirhan E, Horzum MB, Besoluk S. Smartphone addiction proneness in relation to sleep and morningness-eveningness in German adolescents. Journal of behavioral addictions. 2016;5(3):465–73. Epub 2016/08/09. 10.1556/2006.5.2016.056 ; PubMed Central PMCID: PMCPmc5264414. 27499228 PMC5264414

[35] Lee H, Kim JW, Choi TY. Risk Factors for Smartphone Addiction in Korean Adolescents: Smartphone Use Patterns. Journal of Korean medical science. 2017;32(10):1674–9. Epub 2017/09/07. 10.3346/jkms.2017.32.10.1674 ; PubMed Central PMCID: PMCPmc5592183. 28875613 PMC5592183

[36] Lin YH, Chang LR, Lee YH, Tseng HW, Kuo TB, Chen SH. Development and validation of the Smartphone Addiction Inventory (SPAI). PloS one. 2014;9(6):e98312 Epub 2014/06/05. 10.1371/journal.pone.0098312 ; PubMed Central PMCID: PMCPmc4045675. 24896252 PMC4045675

[37] Kim D, Lee Y, Lee J, Nam JK, Chung Y. Development of Korean Smartphone addiction proneness scale for youth. PloS one. 2014;9(5):e97920 Epub 2014/05/23. 10.1371/journal.pone.0097920 ; PubMed Central PMCID: PMCPmc4029762. 24848006 PMC4029762

[38] Choi SW, Kim DJ, Choi JS, Ahn H, Choi EJ, Song WY, et al Comparison of risk and protective factors associated with smartphone addiction and Internet addiction. Journal of behavioral addictions. 2015;4(4):308–14. Epub 2015/12/23. 10.1556/2006.4.2015.043 ; PubMed Central PMCID: PMCPmc4712765. 26690626 PMC4712765

[39] Kuss DJ, Griffiths MD. Social Networking Sites and Addiction: Ten Lessons Learned. International journal of environmental research and public health. 2017;14(3). Epub 2017/03/18. 10.3390/ijerph14030311 ; PubMed Central PMCID: PMCPmc5369147. 28304359 PMC5369147

[40] Staksrud E, Ólafsson K, Livingstone S. Does the use of social networking sites increase children’s risk of harm? Computers in Human Behavior. 2013;29(1):40–50. 10.1016/j.chb.2012.05.026.

[41] Rumpf HJ, Achab S, Billieux J, Bowden-Jones H, Carragher N, Demetrovics Z, et al Including gaming disorder in the ICD-11: The need to do so from a clinical and public health perspective. Journal of behavioral addictions. 2018;7(3):556–61. Epub 2018/07/17. 10.1556/2006.7.2018.59 . 30010410 PMC6426367

[42] Ihm J. Social implications of children's smartphone addiction: The role of support networks and social engagement. Journal of behavioral addictions. 2018;7(2):473–81. Epub 2018/06/06. 10.1556/2006.7.2018.48 ; PubMed Central PMCID: PMCPmc6174576. 29865865 PMC6174576

